# Development of an ototoxicity model in the adult CBA/CaJ mouse and determination of a golden window of corticosteroid intervention for otoprotection

**DOI:** 10.1186/1916-0216-43-12

**Published:** 2014-04-24

**Authors:** Vinay T Fernandes, Vincent YW Lin

**Affiliations:** 1 Department of Otolaryngolgy – Head and Neck Surgery, Sunnybrook Health Sciences Centre, University of Toronto, 2075 Bayview Ave, Suite M1-102, Toronto, ON M4N 3M5, Canada

## Abstract

**Objective:**

To investigate the effect of timing of dexamethasone administration on auditory hair cell survival following an ototoxic insult with kanamycin and furosemide.

**Study design:**

Controlled experimental study.

**Setting:**

Translational science experimental laboratory.

**Methods:**

5–6 week old CBA/CaJ mice, divided into 6 groups, were injected with kanamycin (1 mg/g SC) followed by furosemide (0.5 mg/g IP). Dexamethasone (0.1 mg/g IP) was injected at either 1 hour prior to insult, +1 hr, +6 hr, +12 hr, or +72 hr post insult. Temporal bones harvested on day 7 underwent *Organ of Corti* dissection. Immunohistochemical staining was performed using antibodies to myosin 7a, phalloidin, and TO-PRO.

**Results:**

Hair cell counts demonstrate a uniform ototoxicity model with total loss of outer hair cells (OHCs) and near-total loss of inner hair cells (IHCs). The group pre-treated with dexamethasone showed a statistically significant improvement in counts compared to controls (p = 0.004). Counts from the other experimental groups given dexamethasone after the insult were highly variable but demonstrated some apical and middle turn inner hair cell survival.

**Conclusion:**

Treatment of systemic dexamethasone prior to ototoxic insult attenuates hair cell loss in a reliable, novel, ototoxicity model using kanamycin and furosemide in CBA/CaJ mice. Dosing with dexamethasone following ototoxic insult shows promising yet variable response in hair cell survival.

## Introduction

Administration of corticosteroids has been shown to attenuate the ototoxic effects of aminoglycoside in animal models [1-3]. These effects shown *in vivo* have implications in the treatment of cochleovestibular toxicity. Corticosteroids are also a standard treatment modality in multiple otologic conditions such as Meniere’s disease, sudden sensorineural hearing loss and hearing loss secondary to acoustic trauma. The anti-inflammatory effects of the corticosteroids which reduce the severity of the hair cell loss are thought to be paramount in their efficacy in the treatment of these conditions. Corticosteroids are also used during cochlear implant surgery to minimize the effect of electrode insertion trauma. In the era of expanded candidacy criteria where a growing percentage of patients have reasonable residual low frequency hearing, it is important to maximize our ability to minimize trauma to the remaining auditory hair cells and thus preserve residual hearing.

One of the difficulties for the treating physician is that corticosteroid dosing regiments are still quite variable and traditionally in the role of otoprotection, they are given prior to the initiation of treatment. It is not known if there is any role for administrating systemic corticosteroids after the ototoxic drug has been given. In the field of cardiovascular medicine, the treatment of acute myocardial infarction or acute cerebral vascular accident is dictated by the principle of the ‘golden hour’. If treatment is initiated within an hour of symptom onset, the degree of tissue death is significantly reduced and major morbidity is lessened. In the instance of ototoxicity, our question was whether there was a similar window of time for intervention after the delivery of the ototoxic agent. Specifically, after the ototoxic insult is given, is there a ‘golden hour’ of time in which corticosteroid administration will either prevent or minimize hair cell loss? The purpose of our study is to determine whether the benefits of corticosteroid protection can be extended to after the ototoxic drug is given by studying this effect in a novel adult mouse model of ototoxicity.

## Methods

### Mice

Adult male CBA/CaJ mice (Jackson Laboratories, Bar Harbor, Maine, USA) were allowed free access to water and a regular mouse diet and were kept at room temperature under a standard 12 hour light/dark cycle for one week of acclimatization before the experiments. Animals were between 4–5 weeks of age and of approximately 16–23 g bodyweight. All research protocols were approved by the institutional review board at Sunnybrook Research Institute, Sunnybrook Health Sciences Centre, University of Toronto. Animal care was under the supervision of the Sunnybrook Research Institute, Sunnybrook Health Sciences Animal Facility.

### Ototoxin administration

The first injection for each animal was given at the beginning of the mice daily light cycle. Mice were randomly divided into 6 groups. All intervention groups had kanamycin (Sigma Aldrich, Oakville, ON, Canada, Cat. No. F4381-1G) (1 mg/g) injected subcutaneously followed by furosemide (Sigma Aldrich, Oakville, ON, Canada, Cat. No. F4381-1G) (0.5 mg/g) injected intraperitoneal 30 minutes later (T_0_). Animals were then kept under a heat lamp and provided 100% O_2_ until they returned to normal levels of activity. Those showing signs of severe dehydration or other significant illness were euthanized. All animals were monitored by trained animal care technologists supervised by a veterinarian. A total of 71 mice were used.

### Dexamethasone treatment

**Figure 1:**
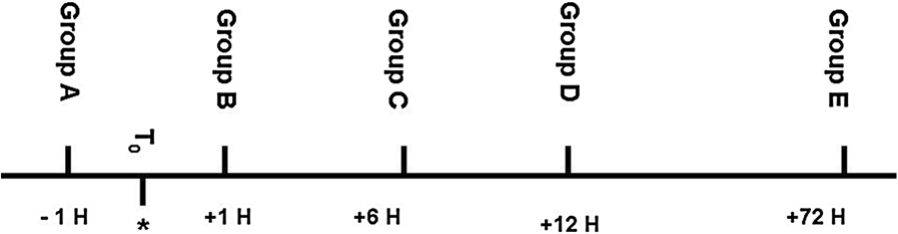
**Study protocol.** *T0 marks the completion of ototoxic insult. First kanamycin is injected followed by furosemide 30 min later, at which point T0 begins. Group A received corticosteroids 1 hour prior to kanamycin injection.

Corticosteroid rescue was administered at varying intervals according to group at a dose of 0.1 mg/g. Group A received dexamethasone (Sandoz, Boucherville, QC, Canada) at T-1 hour prior to ototoxic administration (Figure 1). Group B had dexamethasone administered at T + 1 hour post furosemide injection, Group C had dexamethasone administered 6 hours post furosemide injection, Group D had dexamethasone administered 12 hours post furosemide injection and Group E had dexamethasone administered 72 hours post furosemide injection. The control group had the ototoxic insult administered but no steroid rescue was given, and served as confirmation of the ototoxicity model. All animals were given 1 cc of normal saline subcutaneously on the first day following the ototoxic insult. Mice were anaesthetized with isoflurane and sacrificed by cervical dislocation at 7 days post-corticosteroid rescue or 7 days after the ototoxin regiment in the control group.

### Immunohistochemistry

Immediately following sacrifice, temporal bones were dissected and placed in a 4% paraformaldehyde (Sigma Aldrich, Oakville, ON, Canada, Cat. No. 441244-3KG) solution for 30 minutes then stored in PBS. *Organ of Corti* explants were dissected under a microscope by removing the otic capsule. The remaining tissue, including the stria vascularis, was then dissected away and the remaining tissue containing the cochlear sensory epithelium was cut into apical, second, and basal turns. The cochlear sensory epithelium were permeabilized and blocked in 10% normal goat serum/0.05% Triton X-100 in PBS for 1 hour at room temperature then immediately incubated with primary myosin 7a rabbit Ab (1:250 dilution, Proteus Biosciences Inc., CA, USA, Cat. No. 25–6790) and kept overnight at 4°C. Specimens were then washed in 3 times with PBS, incubated with secondary Cy3 IgG (Jackson Immunoresearch, West Grove, PA) 1:250 dilution in PBS for 2 hours at room temperature, then washed 3 times with PBS. After a final wash with PBS, specimens were incubated with phalloidin 1:250 in 0.05% Triton X-100 PBS (Sigma-Aldrich, Oakville ON, Canada, Cat. No. P5282-1MG), for 30 minutes at room temperature then washed 3 times with PBS. Specimens were finally incubated with TO-PRO (Invitrogen, Burlington, ON, Canada, Cat. No. T3605) 1:500 dilution in PBS for 10 minutes then washed 3 times in PBS. Final triple-labelled specimens were mounted on slides with anti-fade fluorescence mounting media VECTASHIELD® (Vector Laboratories, Burlington ON, Canada). Immuno-labelled surface preparations were imaged with a Zeiss LSM510 confocal microscope (Carl Zeiss MicroImaging GmBH, Germany) equipped with a Spectra Physics multi-line argon laser (Spectra Physics, Santa Clara CA, USA). Confocal settings involved 63 x magnifications with uniform settings throughout all imaging analysis.

### Counting

Samples were analyzed using ImageJ (NIH, USA http://imagej.nih.gov/ij). Inner and outer hair cells were counted for the entire field which was standardized and measured at 146 μm^2^. A cell was considered a hair cell if there was positive myosin VII labelling and the cell was in the appropriate level based upon TO-PRO nuclear labelling. Inner (IHC) and outer hair cells (OHC) were distinguished by their morphology and relative position to their corresponding support cell.

### Statistical analysis

Data was statistically evaluated by using SPSS® (V20, IBM Corp ©). Dependent variables were analyzed with univariate or multivariate analysis using LSD post-hoc test.

## Results

### Ototoxicity model

**Figure 2:**
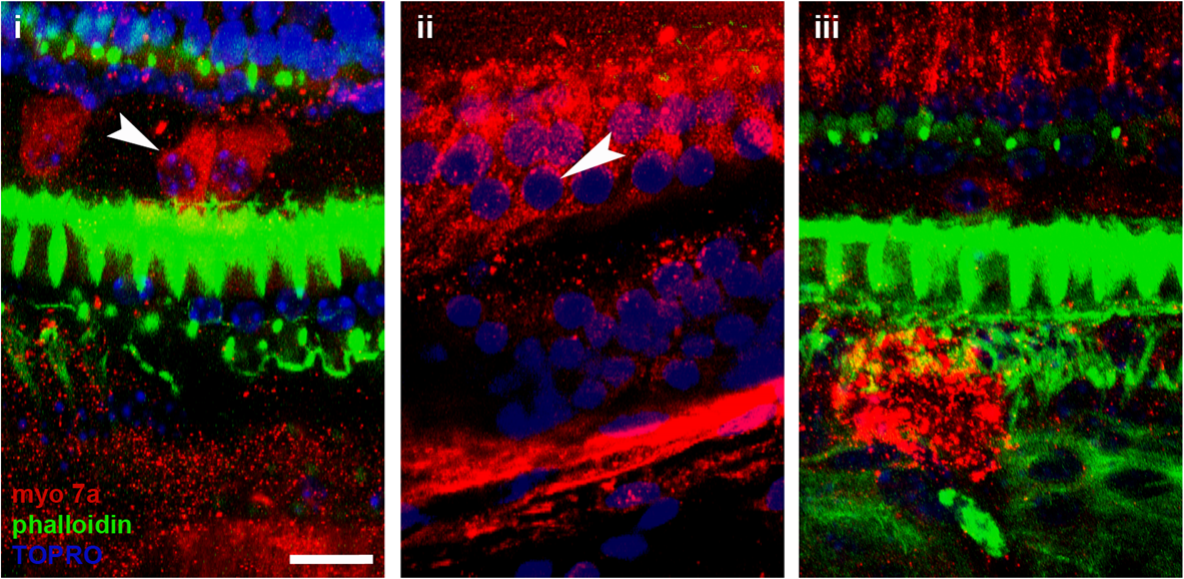
i) Group D, white arrow is pointing to inner hair cell, white scale bar is 10 microns ii) group A, white arrow pointing to inner hair cells, no outer hair cells iii) group F (control), no hair cells seen.

The combined kanamycin and furosemide protocol in CBA/CaJ mice produced a uniform ototoxicity model of hair cell loss. Amongst all control group mice (n = 9), there was no OHC survival in either the basal, second turn, or apical segments (Figure 2iii). We identified total loss of both outer and inner hair cells in the basal turns, as well as near total loss of IHCs amongst second and apical turns. Only 2/9 specimens retained some IHCs in the second turn (average: 2.86 cells/146 μm^2^) and 3/9 specimens retained IHCs in the apical segments (average: 1.56 cells/146 μm^2^). Out of the 71 used in our experiment, 42 survived for tissue analysis for a survival rate of 59.1%.

### Hair cell counts

Viable samples available for analysis on slides included 6 mice from Group A, 7 mice from Group B, 4 mice from Group C, 7 mice from Group D, 7 mice from Group E, and 7 mice from Group F and average hair cell counts are displayed in Table 1.

**Table 1:** Average hair cell counts per standard field

		**Group A (−1 Hr prophylactic)**	**Group B (+1 Hr)**	**Group C (+6 Hr)**	**Group D (+12 Hr)**	**Group E (+72 Hr)**	**Control**
Basal turn	IHC	7.83	0	0	2.5	2.5	0
2^nd^ turn		6.17	5.20	3.5	0.17	5.43	2.86
Apical turn		5.67	0.4	11.75	2.5	9.83	1.56
Basal	OHC	16.33	0.43	0	0	0.25	0
2^nd^ turn		2.17	1.20	0	0	0	0
Apical turn		1	0	0.25	0.25	0	0

**Figure 3:**
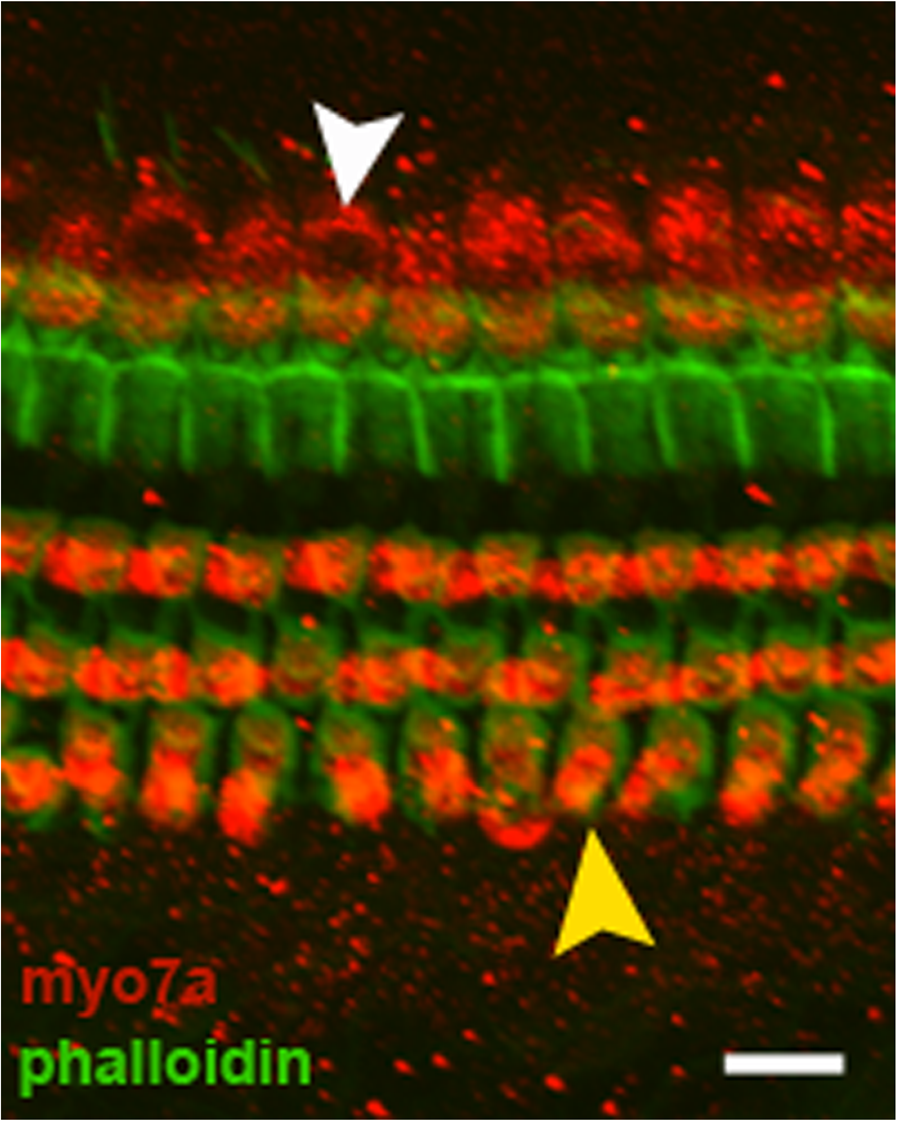
**Normal CBA/CaJ mice.** White arrow indicates normal inner hair cells (IHC). Yellow arrow indicates normal outer hair cells (OHC). White bar represents 10 microns.

**Figure 4:**
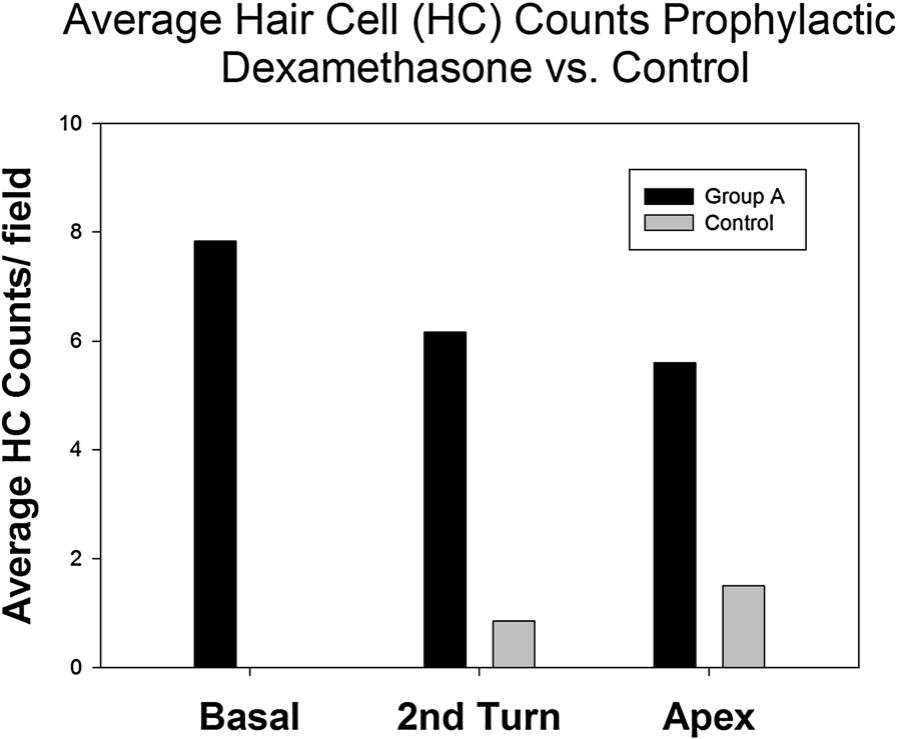
**Comparison of hair cell counts between control group and group A given dexamethasone 1 hour prior to ototoxic insult.** *indicates a statistically significant difference.

A specimen not treated with the ototoxin regimen labelled with myosin 7a and phalloidin depicts 3 rows of OHC and 1 row of IHC in a uniform pattern (Figure 3). In all experimental groups receiving the ototoxin regimen, hair cell loss was profound. In fact, only group A demonstrated OHC survival (Figure 2ii). Group A counts were significantly different from control groups for the basal (F =13.59, p = 0.004) and second turn (F = 5.401, p = 0.04) but not the apex segments (F = 3.22, p = 0.096) (Figure 4).

**Figure 5:**
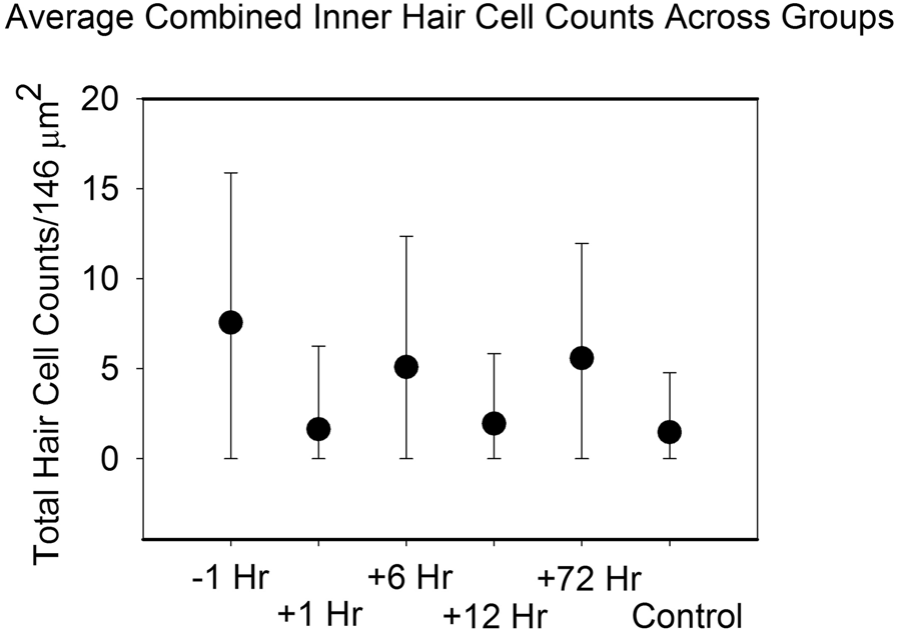
Average inner hair cell counts combining all samples.

Counts across groups B to E were variable and demonstrated no significant pattern amenable to analysis. Almost no OHCs survived despite dexamethasone across groups. There was variable response of at most 2 OHCs in 1–2 specimens per group surviving. IHC survival was also variable, but almost negligible amongst basal segments. Middle and apical segments were further studied. Univariate analysis confirmed significant differences between control and both Group C (p=0.033) and Group E (p=0.019), but not between Group C and Group E themselves (p=0.908). Group D (+12 hr) paradoxically had lower hair cell counts than groups C and E, with only a few hair cells surviving (Figure 2i). No other differences were found between other time point groups and control in terms of OHC survival, or IHC survival in the basal or second turn levels, including when all HC counts were combined and compared across groups (Figure 5).

## Discussion

The otolaryngologist - head & neck surgeon routinely faces an uncertain decision point following consultation for acute cochleovestibular toxicity. For example, effective aminoglycoside therapy in our inpatient units has well known side effects, including cochlear and vestibular toxicity thought to be irreversible and permanent. Exposure to sufficient concentrations causes loss of sensory auditory or vestibular hair cells usually resulting in permanent hearing loss or vertigo. The otolaryngologist is then asked whether intervention is indicated in the acute stage after the drugs are given to minimize or reverse the damage. Further related clinical scenarios involve patients with sudden sensorineural hearing loss presenting to our emergency room. If the patient presents late to the ER, such as several days following the initial recognition of hearing loss, the question will often be asked as to whether steroids should still be recommended. For both these scenarios, there is no research that has examined the effect that timing of administration has on attenuating hair cell loss.

Aminoglycosides have long been known to have ototoxic effects. Some, like gentamicin, have a more vestibulotoxic profile, while others such as kanamycin have a more cochleotoxic profile, which is one of the reasons it was chosen for our experiments. Currently, aminoglycosides are used in auditory research to eradicate hair cells in studies of hair cell regeneration [4]. This class of drugs primarily targets the basal hair cells with a preference for outer hair cells versus inner hair cells. Other effects on the cochlea include clumping of stereocilia, bleb formation, and diminished glycocalyx [5,6]. Although the precise mechanism of aminoglycoside induced auditory hair cell loss is unclear, there is evidence that the formation of reactive oxygen species induces hair cell apoptosis [7,8]. Aminoglycosides can trigger free radial formation that mediates cellular damage. Long-term aminoglycoside administration is further associated with extension of the loss towards the apical hair cells and/or inner hair cell loss.

While aminoglycoside alone has been shown to induce hair cell loss in many animal models, murine ototoxicity models have been shown to be highly ineffective if using aminoglycosides alone [9]. In fact, the dose of kanamycin required to produce ototoxic effects are much higher than in rats and guinea pigs, for reasons that might include pharmacokinetics, bioavailability and activation of the drug. However, synergism with furosemide has been shown to increase the toxic effect, and create more reliable models of hair cell loss [10,11]. The majority of established models to our knowledge involve daily injections over multiple days. The mechanism by which the loop diuretics exacerbate aminoglycoside-induced hearing loss and HC damage is unclear. However, one of the leading hypotheses, given the inhibition by loop diuretics of Na/K/2CL co-transporters, involves a drop in the endocochlear potential that causes a loss of the electrochemical gradient that drives transduction current through hair cells, increasing hearing thresholds and increasing their susceptibility towards apoptosis [12].

Our study employed dexamethasone as a rescue agent. Administration of dexamethasone has been well documented in the attenuation of hair cell apoptosis in animal models, particularly in guinea pig models of apoptosis [13,14]. Himano demonstrated that local administration of dexamethasone directly to the inner ear preceding aminoglycoside administration attenuated hair cell loss. Dexamethasone acts primarily as a glucocorticoid rather than mineralocorticoid, which modulates inflammation and immune responses, and locally increases cochlear blood flow [15]. The efficacy of its otoprotection is related to dosing regimens [16]. Thus, we used very high doses of dexamethasone in our experiments in order to control and maximize our dose response. However, as a result, the doses are not generalizable across species. Nevertheless, previous findings have confirmed that high dose systemic administration resulted in strong dexamethasone labelling of hair cells [17]. Although our high dosing regimen eliminated a dose–response effect, further studies of the effect of timing with varying doses and routes administration of dexamethasone would be extremely valuable.

### Ototoxic regiment is highly effective in ablating both inner and outer hair cells

This paper examines the effect of timing of steroid administration following ototoxic insult. We present a robust ototoxicity model using kanamycin/furosemide to establish consistent near complete OHC loss and high IHC loss in the CBA/CaJ mouse using a newly described single day injection model. Our objective was to establish a rapid systemic protocol for elimination of sensory hair cells in adult mice. This regimen was based on experiments done in other mammals combining aminoglycosides and a loop diuretic [18]. This technique was further described in CBA/CaJ + Swiss-Webster murine model by Oesterle (2008) [19] and validated in a C57BL/6 murine model by Hartman (2009) [20], both of whom used doses of kanamycin (1 mg/g) and furosemide (0.4 mg/g). While these protocols describe total OHC loss similar to our findings, both protocols report IHCs to be largely intact. Taylor employed a similar single injection model in CBA mice using kanamycin with the loop diuretic bumetanide, and showed via aminoglycoside tracers that kanamycin does in fact enter IHCs [21]. The IHC survival rate in this model was still relatively high at 50%. Our model uses a higher variant dose of furosemide (0.5 mg/g) and in an adult CBA/CaJ murine model that establishes a rapid, robust method of destroying both outer and inner hair cells. In fact, we achieved total (100%) IHC loss in 2/3 of control specimens, with the remaining 1/3 only retaining up to 30% of IHCs. This consistency of hair cell loss provides a more reliable and sensitive model that can be used to detect small levels of otoprotection. This model translates clinically only to those patients that demonstrate severe symptoms and effects of ototoxicity and likely represents a small percentage of patients that suffer ototoxicity. The majority of these patients likely have milder symptoms and effects. We decided to not aim for a smaller damage model due to the potential for highly variable results in the control damage group and the reduction in sensitivity that it would provide for our corticosteroid otoprotection regiment.

### Prophylactic treatment with corticosteroids protects mainly inner hair cells and basal outer hair cells

Mice treated with dexamethasone prior to the ototoxic insult had significantly higher cell counts in their basal and second turns. There were large differences between the prophylactic group (Group A) and control group (Group F) that were not as extreme as with the other groups. Paradoxically, there was no statistical difference found in the apical layers, however a clear trend toward greater survival in the prophylactic group was present. It is unclear why we did not see greater differences in the apical segment as expected. Aminoglycosides have been shown in animal studies to be in the stria vascularis and spiral ligament soon after administration, and accordingly thought to enter the cochlea through the vasculature [22]. It may be that a uniform concentration of aminoglycosides within the cochlea enters via the cochlear vasculature interacting with a decreasing basal to apical gradient of dexamethasone uptake demonstrated in our laboratory [17] in a previous study allows for any protective corticosteroid effect to be only detectable in the basal and second turns.

### Corticosteroid rescue is effective in some groups but its effect is highly variable

Counts of hair cells in the *Organ of Corti* following steroid rescue at varying intervals following ototoxic insult demonstrate a protective effect of prophylactic administration of dexamethasone as well a trend toward IHC protection in the apical turn but there was large variability within different treatment groups so trends can be seen but clear patterns cannot be established. However, the preservation of some apical IHCs in groups C and E, and middle turn IHCs in groups B,C and E despite the corticosteroids administered *after* the ototoxic insult demonstrates the potential, albeit, variable potential corticosteroids offer in protecting auditory hairs from undergoing apoptosis.

### Limitations of study

While we have used auditory hair cell counts as a marker for cochlear function, there are limits to this model. We did not assess the status of the auditory nerve or measure function via either evoked brainstem responses (ABR) or otoacoustic emissions (OAE). It would have been helpful to assess the viability of hair cells at earlier time points rather than simply 7 days post corticosteroid administration. In fact Himeno’s work demonstrated that dexamethasone allowed for hair cell protection but had little effect on hearing preservation [2]. Yet, when damaged hair cells initiate the process of apoptosis, the process has been shown in animal models to be attenuated by corticosteroid administration. Therefore we can infer that higher hair cell counts than expected following ototoxin administration indicate a protective effect of steroids in at least one part of the auditory pathway, the *Organ of Corti*.

CBA/CaJ mice have limited genetic variation as they are inbred. Further, they have been shown to have increased susceptibility to kanamycin compared to other murine inbred models such as C57BL6 mice, particularly with age. Accordingly, likely only little of the variability in our results can be explained by genetic variation.

Only prophylactic treatment with corticosteroids prior to ototoxic insult was demonstrated to have any consistent effect in achieving auditory hair cell survival. Our study employed an ototoxic model that resulted in total hair cell loss in the basal segments of mice, as well as near total hair cell loss in the second turn and apical segments. Given the severe loss, our findings are consistent with what we know of clinical scenarios such as those patients who present with sudden sensorineural hearing loss. Poor prognostic factors include severity of loss, suggesting that if the insult is severe enough there may be a threshold that once crossed cannot be reversed. Similar situations exist for brain injury, myocardial infarction and hypoxic injury. Future studies are needed to explore the timing effect of therapy on an ototoxicity model that imparts incomplete yet reliable of hair cell loss which is the equivalent to those patients with a partial threshold shift on auditory testing. These types of patients are more common than those initially presenting with a complete sensorineural loss.

## Conclusion

The administration of kanamycin and furosemide at a single time point provides a reliable ototoxicity model in CBA/CaJ mice. Prophylactic treatment with dexamethasone prior to ototoxic administration attenuates hair cell loss. There is some evidence that the timing of administration of steroid rescue influences hair cell survival but the high degree of variability makes definitive conclusions challenging.

## Competing interests

The authors declare that they have no competing interests.

## Authors’ contributions

VTF carried out the animal experiments, performed all temporal bone dissections, all the immunohistochemical staining, confocal microscopy imaging. Both VTF and VYWL were involved in study design and drafting the manuscript. Both authors read and approved the final manuscript.
